# Editorial: Epistemic Feelings: Phenomenology, Implementation, and Role in Cognition

**DOI:** 10.3389/fpsyg.2020.606046

**Published:** 2020-10-27

**Authors:** Eric Dietrich, Chris Fields, Donald D. Hoffman, Robert Prentner

**Affiliations:** ^1^Department of Philosophy, Binghamton University, Binghamton, NY, United States; ^2^Independent Researcher, Caunes-Minervois, France; ^3^Department of Cognitive Science, University of California, Irvine, Irvine, CA, United States

**Keywords:** belief, knowing, familiarity, agency, confidence

Epistemic feelings, including feelings of familiarity, knowledge, belief, confidence, doubt, confusion, curiosity, agency, ownership, and many others, are ubiquitous components of human experience (Arango-Muñoz, 2014; Proust, 2015). Such feelings are distinct from canonical emotions, yet appear to play similar motivational and regulative roles in cognition, particularly in deliberative, reportable, “process-2” cognition (Evans, 2008; Evans and Stanovich, 2013; see Melnikoff and Bargh, 2018 for criticism of the dual-process distinction). They are the experienced and reportable “propositional attitudes” of folk psychology and pre-computational cognitive representationalism (Fodor, 1978).

Despite their phenomenological ubiquity and historical importance, epistemic feelings are rarely treated as a coherent class of experiences, and relatively little is known about their implementation or their motivational or regulatory roles. Consider, for example, the development of Theory of Mind (ToM) research since Premack and Woodruff (1978). When and how beliefs are attributed to others and the effects of such attributions on the behavior (including verbal reports) of the attributor have been intensively investigated, particularly in children as they develop ToM abilities and in children and adults with impaired ToM abilities (for reviews, see Carlson et al., 2013; Singer and Tusche, 2014). How the “feeling of believing” is generated, however, and how it differs at the neural implementation level from the “feeling of knowing” remain unclear. From a phenomenological perspective, believing and knowing differ in, among other things, felt confidence. While feelings of confidence appear to involve activity in the insulate—cingulate—medial-prefrontal loop (Craig, 2009; Dajani and Uddin, 2015), how they exert their regulatory functions, and how the regulatory roles of “belief” and “knowledge” differ, similarly remain unclear.

Despite the relative lack of investigation of epistemic feelings as a class, considerable information is available about some epistemic feelings considered in isolation. The implementation and functional consequences of feelings of familiarity, for example, have been extensively studied in the visual modality (e.g., Yonelinas et al., 2010). Feelings of confidence in perceptual judgments have been shown to overestimate evidence for the chosen interpretation by discounting evidence for alternatives (Peters et al., 2017). Feelings of agency and ownership have been associated with the medial prefrontal “reality monitoring” system, in part due to their common disruption in schizophrenia (e.g., Simons et al., 2017). Feelings of autonoetic certainty have been mapped to insular cortex in part due to their prevalence in insular-cortex seizures (e.g., Gschwind and Picard, 2016).

The papers in this Research Topic exhibit the eclectic nature of the field. The single experimental paper, by Vogl et al. replicates and extends findings from an earlier study (Vogl et al., 2020) of associations between epistemic feelings (surprise, curiosity, and confusion) and emotions (pride and shame) following successful or unsuccessful completion of a task. They show, in particular, that the epistemic feelings investigated, but not the corresponding emotions, correlate with and possibly motivate knowledge seeking. Cornwall and Higgins review evidence suggesting that epistemic feelings of knowledge (truthfulness) and agency (ability to control) combine with moral feelings to motivate moral judgments and decision making. Perrin et al. consider a particular aspect of autonoetic awareness, the feeling of pastness, and propose that this epistemic feeling contributes to “tagging” some experiences as experiences of a personal past, i.e., as episodic memories. Chang et al. adopt a more abstract approach to experience, arguing from information-theoretic assumptions that events are only experienced (i.e., are only reportably conscious) if their neural implementation is informationally closed, i.e., is sufficient to predict its own future state from its current state as well as that of its environment. In this model, it would be natural to consider epistemic feelings as experienced indicators of predictability, a view compatible with that of predictive-coding models (e.g., Clark, 2013). Levin takes an even bolder step, arguing from considerations of cell and developmental biology that the concepts of “self” and “agency” can and should be extended to the levels of individual cells and functionally-coherent tissues. In this model of “scale-free cognition,” epistemic feelings are components of actionable representations of the environmentally-embedded self not just at the individual-organism scale, but at both smaller and larger scales as well.

In a critical review of the role of “factors” and models across psychology, Jolly and Chang (2019) argue that formalized, quantitative, high-dimensional models capable of integrating data from multiple experiments are needed to move psychology forward. Even in its current, fragmentary state, our understanding of epistemic feelings suggests that they will play central roles in such models. Questions that appear empirically tractable include the following:

- What, in general, is the relationship between epistemic feelings and emotions? The work of Vogl et al. provides a model for addressing this question; how far can such techniques be extended? How are associations between epistemic feelings and emotions implemented? Are the consequences of dissociation pathological, as they appear to be in, e.g., Capgras syndrome (Hirstein and Ramachandran, 1997)?
- As noted earlier, epistemic feelings are most evident during canonical process-2 cognition. What is their role in process-1 cognition, and how do these roles relate? Fields and Glazebrook (2020) have proposed that in the context of a global neuronal workspace model (e.g., Dehaene et al., 2014), subjective probabilities reported during process-2 cognition can be understood as outputs of process-1 cognition. Does this relationship between reportable epistemic feelings and underlying automated processes generalize?
- What is the role of epistemic feelings in predictive processing models? Seth and Tsakiris (2018) have argued that the feeling of being an embodied self that is continuous through time is an experiential correlate of an interoceptive predictive-processing loop. Does this correlation generalize? How does it relate to the feeling of “pastness” studied by Perrin et al.?

As questions such as these begin to be answered, the role of epistemic feelings in both motivating and regulating cognition will become clearer. We expect that this role will prove to be a central one.

## Author Contributions

All authors listed have made a substantial, direct and intellectual contribution to the work, and approved it for publication.

## Conflict of Interest

The authors declare that the research was conducted in the absence of any commercial or financial relationships that could be construed as a potential conflict of interest.
